# Interplay between septins and ubiquitin-mediated xenophagy during *Shigella* entrapment

**DOI:** 10.1080/27694127.2023.2213541

**Published:** 2023-05-17

**Authors:** Damián Lobato-Márquez, José Javier Conesa, Ana Teresa López-Jiménez, Michael E. Divine, Jonathan N. Pruneda, Serge Mostowy

**Affiliations:** a Department of Microbial Biotechnology, National Center of Biotechnology CSIC, Darwin Street, Madrid, Spain; b Department of Structure of Macromolecules, National Center of Biotechnology CSIC, Darwin Street, Madrid, Spain; c Department of Infection Biology, London School of Hygiene and Tropical Medicine, London, United Kingdom; d Department of Molecular Microbiology & Immunology, Oregon Health & Science University, Portland, OR, USA

**Keywords:** autophagy, cryo-SXT, cytoskeleton, septins, *Shigella*, ubiquitin

## Abstract

Septins are cytoskeletal proteins implicated in numerous cellular processes including cytokinesis and morphogenesis. In the case of infection by *Shigella flexneri*, septins assemble into cage-like structures that entrap cytosolic bacteria targeted by autophagy. The interplay between septin cage entrapment and bacterial autophagy is poorly understood. We used a correlative light and cryo-soft X-ray tomography (cryo-SXT) pipeline to study septin cage entrapment of *Shigella* in its near-native state. Septin cages could be identified as X-ray dense structures, indicating they contain host cell proteins and lipids consistent with their autophagy links. Airyscan confocal microscopy of *Shigella*-septin cages showed that septins and lysine 63 (K63)-linked ubiquitin chains are present in separate bacterial microdomains, suggesting they are recruited separately. Finally, Cryo-SXT and live-cell imaging revealed an interaction between septins and microtubule-associated protein light chain 3B (LC3B)-positive membranes during autophagy of *Shigella*. Collectively our data present a new model for how septin-caged *Shigella* are targeted to autophagy.

*Shigella flexneri* is a human-adapted bacterial pathogen and world-leading agent of bacillary dysentery, causing ~160 million illness episodes per annum. *S. flexneri* survives the acidic environment of the stomach and invades colonic epithelial cells, in which bacteria cause local inflammation and tissue damage. After invasion, *S. flexneri* breaks the phagocytic vacuole and gains access to the host cell cytosol, where bacteria can replicate and polymerize host cell actin for actin-based motility and cell-to-cell dissemination. Infected cells can restrict *S. flexneri* replication and dissemination by employing several cell-autonomous immune responses, including antibacterial selective autophagy, also referred to as xenophagy. For ~20 years *S. flexneri* has served as a paradigm to advance our fundamental knowledge of xenophagy, and also how cytosolic bacteria can avoid this cell-autonomous immune response.

In addition to xenophagy, host cells possess other cell-autonomous immune responses to restrict *S. flexneri* infection, including septin cage entrapment. Septins are a poorly understood component of the cytoskeleton that interact with membranes to form nonpolar filaments and higher order assemblies, such as rings and cage-like structures. By recognizing micron-scale membrane curvature, septins play key roles in a variety of cellular processes including cytokinesis, phagocytosis and mitochondrial fission. In the cytosol, septins can entrap actin-polymerizing *S. flexneri* in cage-like structures that restrict its actin-based motility. Septin cage-entrapped *S. flexneri* are targeted to destruction by xenophagy, highlighting the septin cage as a unique opportunity to investigate the role of cytoskeleton in cargo selection during selective types of autophagy. The machinery underlying xenophagy can be recruited via ubiquitylation. Ubiquitin is a small protein that post-translationally modifies proteins and bacterial lipid A, an essential component of the outer membrane of Gram-negative bacteria. Ubiquitin contains 7 lysines and an N-terminal methionine residue that can be covalently linked to another ubiquitin monomer; this feature allows ubiquitin to modify targets with different polyubiquitin lengths and linkages that direct distinct signaling outcomes. For example, it is well known that lysine 63 (K63)-linked and lysine 48 (K48)-linked polyubiquitin chains target cargos to autophagic and proteasomal degradation, respectively. Selective autophagy receptors such as Sequestosome 1 (SQSTM1)/p62 or Calcium-binding and coiled-coil domain 2 (CALCOCO2)/NDP52 can bind ubiquitin and also recruit microtubule-associated protein light chain 3B (LC3B), a historical component of the canonical autophagy machinery. Despite investigation of the *S. flexneri* septin cage for almost 15 years, the coordination of septin cage entrapment and ubiquitin-mediated xenophagy was mostly unknown.

In new work [1], we employed cutting-edge microscopy approaches to study septin-autophagy interactions during septin cage entrapment of *S. flexneri in situ*. First, we optimized a correlative cryo-light and cryo-soft X-ray tomography (cryoSXT) pipeline to visualize *S. flexneri* septin cages inside human epithelial cells at high resolution. CryoSXT enables imaging of unstained and cryo-preserved biological samples in their near native state, and is highly suited to study membrane-based organelles, including bacteria and autophagosomes. Using correlative cryo-light and cryoSXT we visualized, for the first time, *S. flexneri* septin cages at ~30 nm resolution *in situ* during infection of human epithelial cells. In this case, septin cages could be identified as X-ray dense structures, suggesting the presence of host factors, e.g., proteins and lipids, surrounding entrapped bacteria in addition to septins. We next infected HeLa cells stably producing a Green Fluorescent Protein fused to SEPTIN6 (GFP-SEPT6) and transiently transfected with mCherry-LC3B. Imaging these infected cells by cryoSXT revealed that septins interact with LC3B-positive membranes during xenophagy of *S. flexneri*. In agreement, Airyscan confocal microscopy and live-cell imaging confirmed septin-LC3B-positive membrane interactions during septin cage entrapment, supporting a coordinated recruitment of septins and the autophagy machinery to cytosolic *S. flexneri*.

It was not known which type(s) of polyubiquitin decorate entrapped *S. flexneri*, nor how septins and ubiquitin coordinate the recruitment of autophagy machinery to entrapped bacteria. We discovered that K63, but not K48 polyubiquitin chains, co-localize with septin-caged bacteria. Consistent with a role for K63 polyubiquitin chains in the recruitment of autophagy machinery, we showed that the vast majority of K63-positive *S. flexneri* septin cages also co-localize with LC3B. Surprisingly, Airyscan confocal microscopy revealed that septins and K63 polyubiquitin are present in non-overlapping microdomains on *S. flexneri*. To investigate septin-ubiquitin interplay we employed small interfering RNA (siRNA) to deplete SEPTIN7 (SEPT7) in infected cells. SEPT7 depletion did not affect the frequency of ubiquitylated *S. flexneri*, indicating that recruitment of septins and K63 polyubiquitin chains to bacteria are independent. Quantitative microscopy showed that K63 polyubiquitin chains co-localize with LC3B (but not septins) at the *S. flexneri* septin cage. It also showed that septins co-localize with LC3B (but not K63 polyubiquitin chains), supporting the notion of a role for septins in the recruitment of autophagosomes to entrapped bacteria.

Collectively, our data suggest a new model (Figure 1), where: i) micron-scale membrane curvature promotes septin recognition of the poles of cytosolic *S. flexneri* for cage entrapment; ii) unknown ubiquitin ligase(s) decorate separate regions of the bacterial surface (not covered by septins) with K63 polyubiquitin chains; iii) xenophagy receptors (such as p62, NDP52) link ubiquitylated bacteria to LC3B-positive membranes; iv) septins interact with LC3B-positive membranes to promote autophagosome formation around *S. flexneri*; v) lysosomes fuse with autophagosomes to eliminate septin cage-entrapped bacteria.

**Figure 1.**
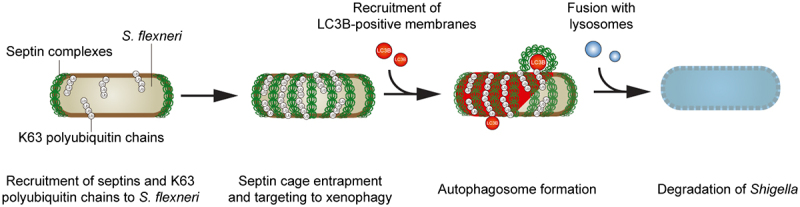
Working model depicting the coordination of septin cage entrapment and antibacterial xenopagy of *S. flexneri*.

Many exciting questions emerge from this work. In our study [1] we discover that K63 polyubiquitin chains are present in microdomains on bacteria distinct from microdomains recognized by septins. In a next step, it will be of great interest to identify the precise targets of septins and K63 polyubiquitin on the bacterial surface. What are the roles of septin and K63 polyubiquitin microdomains during xenophagy of *S. flexneri*? We have previously shown that *S. flexneri* septin cage entrapment can be reconstituted *in vitro* in a cell-free system (in the absence of ubiquitin) using purified recombinant septins. From this, we conclude that ubiquitin is not required for septin assembly into cages, however, we cannot rule out a role for ubiquitin in the regulation of septin cage activity. Finally, what is the precise role of septins in autophagosome formation? We failed to identify a direct interaction between SEPT7 and LC3B during *S. flexneri* infection, but speculate that septins, previously described to play a role in membrane fusion, are important in autophagosome formation. LC3B is one of the most important downstream factors of the autophagy machinery; septin interaction with early components of the cascade of the autophagy machinery, including subunits of the Unc-51-like kinase (ULK) and/or Phosphoinositide 3-kinase (PI3K) complex, or Autophagy-related protein 9A (ATG9A), may be more relevant to have a local induction of autophagosome biogenesis. To address this, we propose that working with septins, ubiquitination and autophagy reconstitution systems *in vitro* will help discover how septins and K63 polyubiquitin coordinate the autophagy of entrapped *S. flexneri*.

## List of abbreviations

SEPT6: human SEPTIN6, SEPT7: human SEPTIN7, LC3B: microtubule-associated protein light chain 3B, P62 or SQSTM1: Sequestosome 1, NDP52 or CALCOCO2: Calcium-binding and coiled-coil domain 2, GFP: Green Fluorescent Protein, K63 polyubiquitin: lysine 63-linked polyubiquitin chains, K48 polyubiquitin: lysine 48-linked polyubiquitin chains, ULK: Unc-51-like kinase, PI3K: Phosphoinositide 3-kinase, ATG9A: Autophagy-related protein 9A.
